# Crystallinity of tellurium capping and epitaxy of ferromagnetic topological insulator films on SrTiO_3_

**DOI:** 10.1038/srep11595

**Published:** 2015-06-30

**Authors:** Jihwey Park, Yeong-Ah Soh, Gabriel Aeppli, Xiao Feng, Yunbo Ou, Ke He, Qi-Kun Xue

**Affiliations:** 1 London Centre for Nanotechnology, University College London, London WC1H 0AH, United Kingdom; 2 Swiss Light Source, Paul Scherrer Institut, Villigen PSI, CH-5232, Switzerland; 3 Laboratory for Solid State Physics, ETH Zurich, Zurich, CH-8093, Switzerland; 4 Institut de la Matiere Complexe, EPF Lausanne, Lausanne, CH-1015, Switzerland; 5 Department of Physics, Tsinghua University, Beijing 100084, People’s Republic of China; 6 Institute of Physics, Chinese Academy of Sciences, Beijing 100190, P. R. China

## Abstract

Thin films of topological insulators are often capped with an insulating layer since topological insulators are known to be fragile to degradation. However, capping can hinder the observation of novel transport properties of the surface states. To understand the influence of capping on the surface states, it is crucial to understand the crystal structure and the atomic arrangement at the interfaces. Here, we use x-ray diffraction to establish the crystal structure of magnetic topological insulator Cr-doped (Bi,Sb)_2_Te_3_ (CBST) films grown on SrTiO_3_ (1 1 1) substrates with and without a Te capping layer. We find that both the film and capping layer are single crystal and that the crystal quality of the film is independent of the presence of the capping layer, but that x-rays cause sublimation of the CBST film, which is prevented by the capping layer. Our findings show that the different transport properties of capped films cannot be attributed to a lower crystal quality but to a more subtle effect such as a different electronic structure at the interface with the capping layer. Our results on the crystal structure and atomic arrangements of the topological heterostructure will enable modelling the electronic structure and design of topological heterostructures.

In topological insulators (TI) gapless, helical Dirac fermion like surface states reside within a bulk band gap^1^,^2^,^3^,^4^. The surface states are protected by time reversal symmetry and against backscattering from nonmagnetic defects due to spin-momentum locking^5^, which has suggested eventual applications to low-power spintronics and fault-tolerant quantum computation. Furthermore the breaking of time reversal symmetry by introducing ferromagnetism in TI’s can give rise to novel quantum phenomena^6^,^7^,^8^,^9^,^10^ including a quantum Hall effect in the absence of an external magnetic field, which has been realized and confirmed in Cr_*x*_(Bi_y_Sb_1−y_)_2−x_Te_3_ thin films^11^,^12^,^13^.

For applications, it might seem most promising to work with films deposited on silicon substrates, and we have previously shown that high-quality epitaxial Bi_2_Te_3_ films can be grown on Si (111) wafers^14^. Nonetheless, the most promising electrical transport data have been found for SrTiO_3_ substrates^11^, perhaps because of the same polar characteristics that make SrTiO_3_ so popular for oxide heterostructures. It is not only the substrate, but also the capping layer which determines the electrical properties of films. In particular, the surfaces of three-dimensional TI’s are prone to oxidization and environmental doping by exposure to air resulting in degradation of the surface characteristics^15^,^16^,^17^,^18^. Protection of the TI surface from such degradation is, therefore, crucial to realize TI-based electronics devices. One method used widely for the protection of the TI surface is the deposition of an insulating layer at the top^19^,^20^,^21^,^22^, which can also provide a gating tool to control the chemical potential of the TI^21^,^23^,^24^, but its structure and influence on the properties of the TI is little known^25^.

Tellurium is an attractive choice for a capping layer since it can be deposited in UHV soon after the growth of TI films. It has been shown to be insulating enough for gating TI films and does not require to be removed. For example, we have grown Al_2_O_3_ layer at 100 °C with atomic layer deposition (ALD) on Te capped (Bi,Sb)_2_Te_3_ TI films and obtained good top-gate devices. We have found that without the Te capping layer the top-gate device shows lower quality due to oxidation of the films during ALD. On the other hand, thermal stability is not very good since Te starts desorbing at around 200 °C. However, depositing gate or contact layers at 100 °C has shown no detectable influence on the Te capping layer. While the Te capping layer does not influence much the usual transport properties (mobility, carrier density) of the TI it has been, in particular, reported that it hinders the occurrence of the quantum anomalous Hall effect in Cr doped (Bi_x_Sb_1−x_)_2_Te_3_ (CBST) films^11^ but no systematic study has been carried out.

Here we investigate the effect of a Te capping layer on the CBST thin film by x-ray diffraction (XRD) and reveal that not only the CBST film but also the Te capping layer grows epitaxially and the deposition of the capping layer does not degrade the crystallinity of the CBST film. In addition, we observe that exposure to x-rays causes the sublimation of CBST, which is prevented by the Te capping layer.

## Results

**Figure 1:**
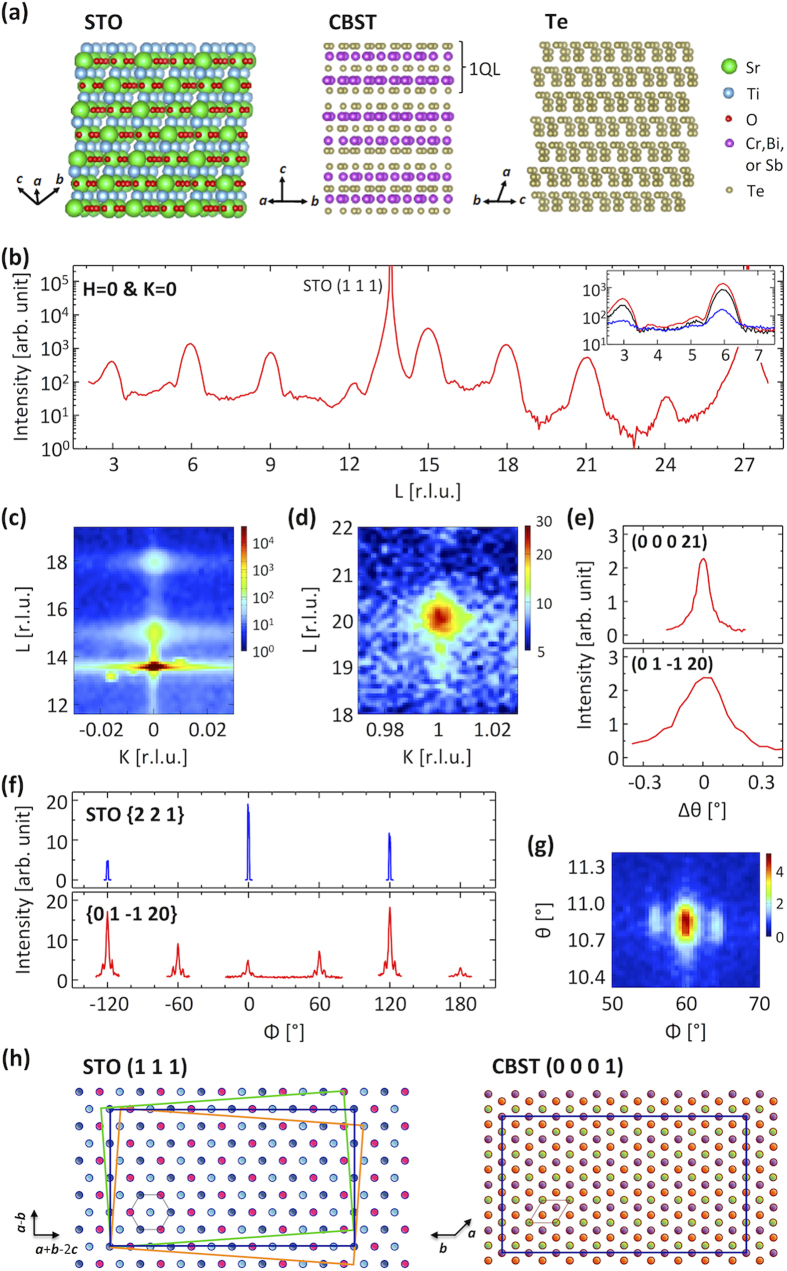
XRD measurements for the uncapped CBST film. (**a**) The schematic crystal structures of STO, CBST, and Te. (**b**) XRD pattern along the surface normal direction. All of the peak positions correspond to (n n n) of the cubic STO lattice or (0 0 0 3n) of the hexagonal CBST lattice. (Inset) XRD results measured with the same conditions at various time points. Red, black, and blue lines are measured after the exposure to x-rays for about 5, 9, and 21.5 hours, respectively. (**c,d**) Reciprocal space maps in the K-L plane with H = 0. Sharp spots at positions only satisfying the space group of STO or CBST lattice confirm the epitaxial growth of the CBST film. (**e**) Rocking curves for CBST (0 0 0 21) and (0 1 –1 20) reflections. The width of the (0 1 –1 20) peak is wider than that of (0 0 0 21) indicating better orientational order along the stacking direction. (**f**) Azimuthal scans of STO {2 2 1} and CBST {0 1 –1 20} reflections in which the azimuthal angle is defined with respect to the [1 1 –2] and [0 1 –1 0] directions on the surface of STO and CBST, respectively. The relation between the azimuthal peak positions indicates an in-plane epitaxial relationship of STO [1 1 –2] || CBST [0 1 –1 0]. (**g**) θ-Φ map of the (0 1 –1 20) reflection. Two satellite peaks separated by about 4° from the main (0 1 –1 20) peaks are present indicating three crystal domains where the crystallographic axes of the satellite domains are tilted by about ±4°. (**h**) Schematic diagram of the supercells showing a reasonable lattice match. The different colored spheres represent metal atoms, making no distinction between different kinds of atoms, in different planes with oxygen atoms being ignored. The cell presented by blue rectangles has a size of around 66 × 38 Å^2^. Green and orange rectangles rotated by 4° from the blue rectangle have (lower left to upper right) diagonal vortices on different colored spheres, i.e., on different planes.

The crystal structures of the constituents of the films are depicted in Fig. 1(a). SrTiO_3_ (STO) has a perovskite structure composed of a periodic stacking of Ti and SrO_3_ planes along the [1 1 1] direction where the distance between nearest Ti or Sr atoms on the (1 1 1) plane is 5.523 Å. CBST is a tetradymite-type crystal structure with lattice constants of *a* ~ 4.3 Å and *c* ~ 30 Å and the crystal structure of Te is hexagonal with lattice constants of *a* = 4.456 Å and *c* = 5.921 Å. Therefore STO, CBST, and Te have large lattice mismatch between one another.

Figure 1 presents x-ray diffraction results for the uncapped sample. In Fig. 1(b), specular diffraction peaks, except at L = 13.6 and 27.2 that originate from the STO substrate, exist only at the positions corresponding to the (0 0 0 3n) (n = integer) reflections with a lattice constant of *c* = 30.62 ± 0.06 Å on the basis of a hexagonal unit cell indicating that the growth direction of the film is along the *c* axis; i.e. the (0 0 0 1) plane of the CBST is parallel to the (1 1 1) plane of the STO substrate. Sharp spots in reciprocal space maps, as seen in the panels (c) and (d), reveal that the film is crystalline with well-defined orientations, in which every spot position of CBST satisfies a space group of R-3m of the tetradymite-type structure. A large difference of the rocking curve widths of specular and asymmetric Bragg reflections is observed, in contrast to Bi_2_Te_3_ films on Si substrates^14^; the full widths at half maximum (FWHMs) of the rocking curves at the (0 0 0 21) and (0 1 –1 20) reflections are 0.08° and 0.27°, respectively, as shown in Fig. 1(e). The in-plane lattice constant obtained from the peak position is ≈4.28 Å. The in-plane epitaxial relationship obtained from the relation of the STO {2 2 1} and CBST {0 1 –1 20} peak positions in their azimuthal scans, presented in Fig. 1(f) where the {0 1 –1 20} reflection has a peak at every 60° due to twin domains^26^, is STO [1 1 –2] || CBST [0 1 –1 0]. Given the above epitaxial relationship, the smallest supercell demonstrating a good lattice match within 1% has a size of about 66 Å × 38 Å that contains 32 Sr or Ti ions on the STO (1 1 1) plane and 54 (Cr,Bi,Sb) or Te ions on the CBST (0 0 0 1) plane, as depicted in Fig. 1(h).

The CBST consists of a stacking of quintuple layers (QLs; see Methods) along the *c* axis, as seen in Fig. 1(a), where the QLs are bonded to each other by weak van der Waals force while the atoms inside the QL are subject to strong covalent bonding. Its directional bonding character allows the film to have good orientational order along the stacking direction despite the lattice match only with a huge supercell. In the plane, the interaction between the film and substrate is sufficient for epitaxial growth but is not strong enough to result in good in-plane orientational order. In addition, the azimuthal scans of the film show two satellite peaks separated by approximately 4° from the main peak indicating that the film has three domains where the crystallographic axes of the satellite domains are misoriented by about ±4°. This misorientation angle is consistent with rotations of the CBST relative to the SrTiO_3_ supercells shown in Fig. 1(h). The atoms of the two supercells clearly do not line up as they do in the unrotated case, which provides a potential explanation as to why the intensities for the supercells are lower when they are rotated. The misorientation angle itself is simply the angle needed to rotate the diagonal of the aligned supercell, where it connects two atoms in the same layer, to a position where it connects two atoms in different layers, suggesting that steps on the surface of the (111) substrate could nucleate the rotated supercells.

While performing the XRD measurement, the intensities of the (0 0 0 3n) reflection and the visibility of the thickness fringes decreased with time, as seen in the inset of Fig. 1(b), which we attribute to the sublimation of CBST caused by x-ray irradiation. Since the region illuminated by x-rays was varying due to the rotation of the sample, the sublimation occurred nonuniformly and therefore strongly suppressed the thickness fringes. The CBST film disappeared from the substrate after exposure to x-rays for about 22 hours, which we verified by visual inspection. X-rays can destabilize Cr, which can induce desorption or formation of new compounds. We speculate that the loss of Cr causes the degradation of the film, which is supported by the fact that Bi_2_Te_3_ films did not undergo degradation when we conducted XRD measurements^14^.

**Figure 2:**
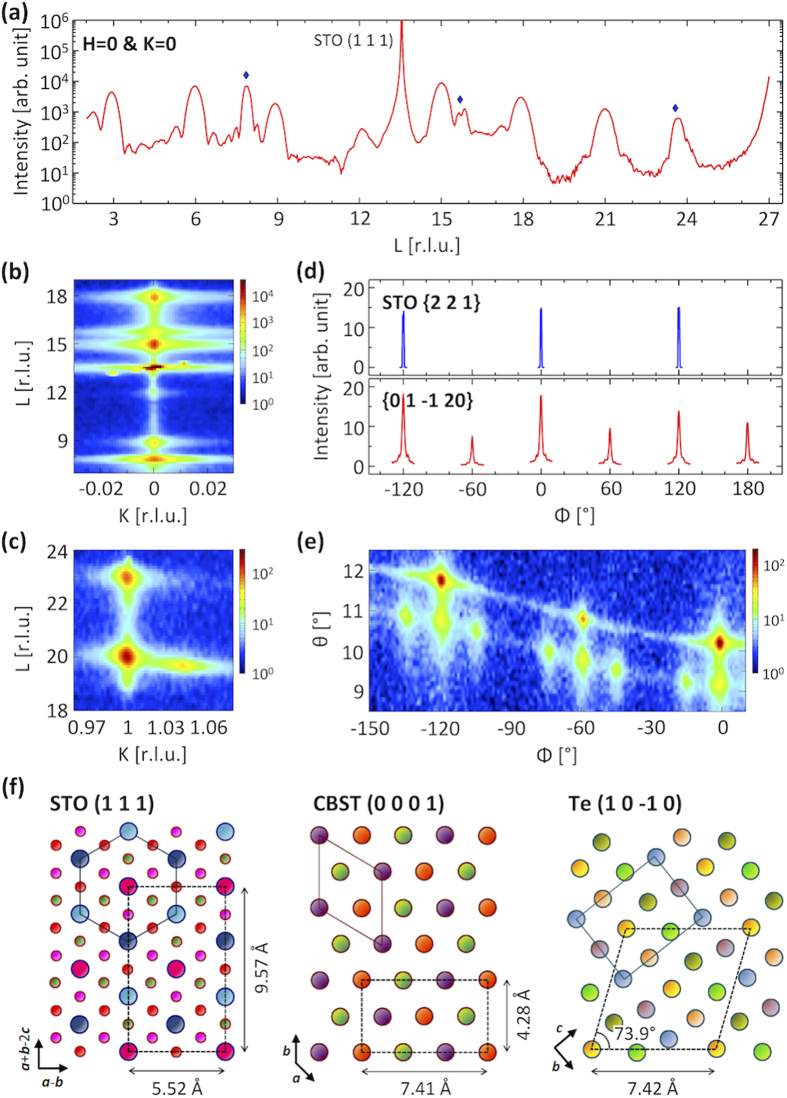
XRD measurements on the CBST film capped by Te. (**a**) XRD pattern along the surface normal direction. All peak positions except three peaks marked with diamond satisfy the space group of CBST or STO and the additional three peak positions are consistent with the (n 0 -n 0) reflection of bulk Te crystal with a lattice constant *a* = 4.47 ± 0.03 Å. (**b,c**) Reciprocal space maps in the K-L plane with H = 0. Sharp spots are present only at positions consistent with STO, CBST or Te lattice indicating that Te as well as CBST is grown epitaxially. (**d**) Azimuthal scans of STO {2 2 1} and CBST {0 1 –1 20} reflections in which the azimuthal angle is defined with respect to the [1 1 –2] and [0 1 –1 0] directions on the surface of STO and CBST, respectively. The capped and uncapped films satisfy the same epitaxial relationship. (**e**) θ-Φ map around the (0 1 –1 20) reflection of CBST. The spots separated by 1° in θ from the CBST (0 1 –1 20) reflections correspond to Te (2 1 –3 1) reflection from which the epitaxial relationship of Te [0 1 –1 1] || CBST [2 1 –3 0] has been obtained. (**f**) The crystal structures of STO, CBST, and Te projected to the planes normal to the stacking direction. The different colored spheres represent atoms regardless of kinds of atoms in different planes; small spheres at left are oxygen atoms. The d-spacing and angle of Te lattice presented were calculated with the lattice constants of bulk Te, which may be slightly different from those of the Te film due to strain.

The sublimation can be prevented by the deposition of a Te layer on top of the TI film. In the film capped with Te no change in XRD results occurred over time. Figure 2(a) shows a reciprocal space scan along the surface normal direction of the capped film. The peaks originating from the CBST film and the STO substrate are present at the same positions as those of the uncapped sample. In addition, three peaks are observed at L = 7.9, 15.8, and 23.7 [marked with diamonds in Fig. 2(a)], which correspond to the (n 0 -n 0) reflection of the hexagonal lattice of Te. The lattice constant obtained from the peak positions is *c* = 30.66 ± 0.19 Å for CBST and *a* = 4.47 ± 0.03 Å for Te, slightly larger than that of bulk Te. The Scherrer equation uncorrected for instrumental broadening, with a Scherrer constant of 0.885^27^, provides the crystalline thickness of 67.2 ± 0.7 Å for CBST and 108.7 ± 1.9 Å for Te, consistent with their nominal thicknesses of 6 nm and 10 nm. Furthermore, the measured ratios of the scattering intensities of the CBST (0 0 0 3n) and Te (n 0 -n 0) reflections are also consistent with a calculation assuming perfect crystalline films of the nominal thicknesses. Therefore, the Te cap is fully crystalline with negligible amorphous inclusions. The azimuthal relation between the CBST and the STO substrate shown in Fig. 2(d) confirms the same epitaxial relationship as the uncapped film.

The large lattice mismatch between CBST and Te and the extremely low growth temperature were expected to result in an amorphous Te layer but the reciprocal space maps reveal, surprisingly, that the Te overlayer as well as the CBST film grew epitaxially with the conventional hexagonal (SG 152) structure of bulk tellurium. The spot at K = 1.04 and L = 19.64 in Fig. 2(c) has a width which is 65% of that at the (0 1 –1 20) spot along the L direction, consistent with the thickness ratio of the Te layer to the CBST layer evidencing that it originates from the Te layer and corresponds to the (2 1 –3 1) reflection of Te. The azimuthal relation between Te (2 1 –3 1) and CBST (0 1 –1 20) reflections presented in Fig. 2(e) gives the epitaxial relationship of Te [0 1 –1 1] || CBST [2 1 –3 0]. The diagonal length of the Te lattice on its (1 0 –1 0) plane (7.42 Å) is well matched to the distance between the second nearest (1 0 -1 0) planes of CBST (7.41 Å), as depicted in Fig. 2(f). This d-spacing match occurs in every case that one of the diagonal directions of the Te lattice is along a* or b* reciprocal axis of CBST with the other diagonal direction off by 14° from the other CBST reciprocal axis. Therefore Te {2 1 –3 1} reflections appear at the same azimuthal angles as CBST {0 1 –1 20} reflections and at positions separated by 14°.

**Figure 3:**
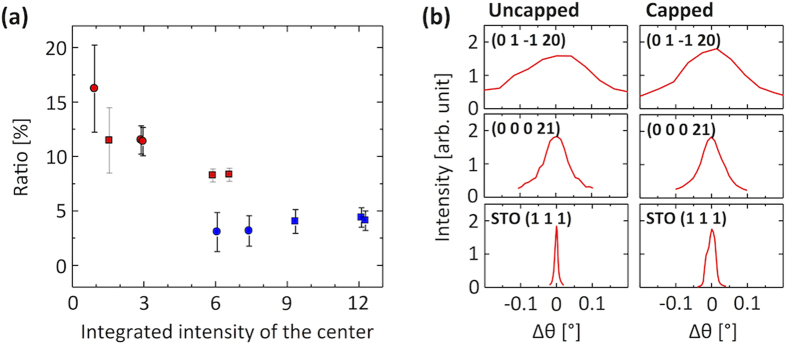
(**a**) The intensity ratio of the satellites to the main peak. Red circles and squares represent the uncapped sample and blue ones the capped film. Circles and squares represent different twin domains. The intensities have been obtained by fitting Lorentzian lineshapes assuming symmetric satellite peaks i.e. both have same height, width, and tilt angle. We also assume the peak widths and the distance between the main and satellite peaks are the same for all the peaks belonging to the same domains where the {0 1 –1 20} reflection appears every 120°. (**b**) Rocking curves of STO (1 1 1) and CBST (0 0 0 21) and (0 1 –1 20) reflections. Note that the widths are similar for both films regardless of the crystalline quality of the STO substrate.

The satellites shown in the azimuthal scan of the CBST {0 1 –1 20} reflections are observed in the capped sample as well, as shown in Fig. 2(d), but their ratios to the main peak are weaker. Figure 3(a) shows the intensity ratio of the satellites to the main peak as a function of the integrated intensity of the main peaks where the intensities have been obtained by fitting Lorentzian lineshapes assuming symmetric satellite peaks, same peak widths and same distance between the main and satellite peaks for all azimuthal angles of the same domain. The intensity variation of the main peaks is due to the presence of twin domains with one domain having a larger population than the other and the oblique scattering geometry exposing different areas of the film as the azimuthal angle is being varied. The smaller intensity of the main peaks for the uncapped film corresponds to more sublimation of CBST and thus the TI texture is more sensitive to the layers immediately adjacent to the substrate. In the capped film the ratio of the satellites to the main peak is almost constant whereas the ratio is higher for the smaller main peak in the uncapped film. The almost constant ratio in the capped films suggests that the film characteristics along the direction parallel to the growth are homogeneous across the whole film. The higher ratio in the uncapped film indicates that the deeper layers closer to the substrate have a larger area of tilted domains, which merge into the main domain as the film becomes thicker, consistent with a previous report^28^.

As seen in Fig. 3(b) the rocking curve widths of both uncapped and capped films are similar for each reflection direction and wider than those of the STO substrates. The crystalline quality of the CBST films is independent of that of the STO substrate, in strong contrast to TI films grown on substrates having a good lattice match with a small cell where the structural quality of the film is locked to that of the substrate for any orientation^17^, which enables the growth of films with very high crystalline quality^28^.

## Discussion

CBST films grown on STO have shown novel electrical transport characteristics^11^ in spite of the relatively poor crystalline quality, supporting the robustness of the topological surface state^1^. On the other hand, addition of a capping layer hinders the observation of sensitive effects such as the anomalous quantum Hall effect. Furthermore, TI films with very high crystalline quality grown on Si substrates did not display anomalous macroscopic electrical transport properties, despite verification of the TI states by surface probes such as scanning tunneling microscopy and angle resolved photoemission spectroscopy (ARPES), which we believe^14^ is due to the doping of the Si substrate by Te.

To provide a further atomistic basis for understanding the variable electrical transport data, we have characterized via XRD the crystal structure of CBST films grown on STO (1 1 1) substrates with and without a Te capping layer. The XRD results reveal that both CBST and Te films grow epitaxially with the epitaxial relationship of STO [1 1 1] || CBST [0 0 0 1] || Te [2 1 –3 0] and STO [1 –1 0] || CBST [2 1 –3 0] || Te [0 1 –1 1]. The orientational order of the CBST film is poorer in the *a*-*b* plane than along the *c* axis, independent of the crystal quality of the STO, which we attribute to the poor lattice matching with large supercells and the directional bonding character of CBST. In addition, severe sublimation of the CBST film by x-rays has been observed, which can be prevented by the deposition of a Te capping layer. Although the deposition of a Te capping layer does not degrade the structural quality of the CBST film and well protects the films from sublimation in x-ray measurements, it does significantly reduce the anomalous Hall resistance of the films below the quantized plateau and therefore the change of its electrical transport properties compared to uncapped films may originate from the different electronic structures of the CBST surface state depending on the adjacent (both capping and substrate) materials^21^. In particular, the Te layer can introduce other conduction channels by band bending at the interface.

**Figure 4:**
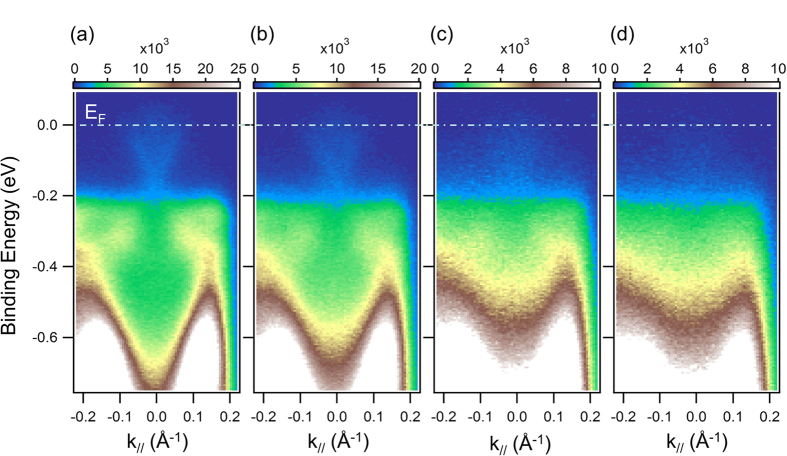
Room temperature angle resolved photoemission spectroscopy (ARPES) data of (Bi,Sb)_2_Te_3_ film with varying tellurium capping thickness. k|| refers to the Κ-Γ-Κ direction in the reciprocal space of (Bi,Sb)2Te3 film. (**a**) uncapped 5 QL (Bi,Sb)_2_Te_3_ film, (**b**) 5 QL (Bi,Sb)_2_Te_3_ film with 0.625 nm Te capping layer, (**c**) 5 QL (Bi,Sb)_2_Te_3_ film with 1.25 nm Te capping layer, (**d**) 5 QL (Bi,Sb)_2_Te_3_ film with 1.875 nm Te capping layer.

To investigate the influence of the Te capping layer on the electronic structure of the TI, we conducted ARPES at room temperature of (Bi,Sb)_2_Te_3_ films grown on Nb-STO(111) with different Te capping layer thickness. To avoid band fading, we used 5 QL (Bi,Sb)_2_Te_3_ films instead of CBST for ARPES measurement. In Fig. 4, we plot the ARPES data of four different samples of 5 QL (Bi,Sb)_2_Te_3_ films: uncapped and capped with 0.625, 1.25, and 1.875 nm thick Te layer. As the Te layer thickness increases, we do not detect obvious changes in the band dispersion or doping level nor do we detect a Te-derived metallic band, but the Dirac surface states of the TI gradually fade as the interface between the TI and capping layer move beyond the probe depth of our VUV ARPES experiments, which is ~1 nm below the surface. It is understandable that we cannot resolve the issue of how the surface states of the TI are affected by the addition of the capping layer using VUV ARPES since the interface becomes buried below the surface as we increase the capping layer thickness.

On the other hand, the knowledge that the capping layer is conventional crystalline tellurium, which is a small gap (~0.3 eV) semiconductor^29^, should enable density functional calculations to determine the actual interface states and the chemical potential which they are subjected to, and especially whether any features of the topological surface states for the uncapped material are inherited by this interface. In such superslab calculations, it will also be important to consider the polar SrTiO_3_ substrate, which seems to be essential for the observation of the quantum anomalous Hall effect; our characterization of the epitaxy relative to the substrates should facilitate this.

## Methods

The Cr_x_(Bi_y_Sb_1−y_)_2−x_Te_3_ (x ~ 0.15, y ~ 0.1) thin films were prepared by molecular beam epitaxy (MBE) in an ultra high vacuum (UHV) system with the base pressure of ~5 × 10^-11^ mbar. MBE growth was performed by co-evaporating Bi (99.9999%), Sb (99.9999%), Cr (99.9999%) and Te (99.9999%) from standard Knudsen cells onto commercial SrTiO_3_ (1 1 1) substrates kept at ~180 °C. The CBST film thickness is around 6 nm, which is 5 nm of continuous film with additional 1–2 nm height islands on top, confirmed by atomic force microscopy (AFM) images of samples grown under the same parameters as the films investigated in this study. For XRD measurements, the extra islands contribute to the intensity. If the coverage of the extra islands is over 50% then the thickness deduced from the XRD measurements will be around 6 nm consistent with our results. A Te capping layer with the thickness of ~10 nm was deposited by MBE at ~150 K onto a film before we took it out of the UHV chamber for measurements. CBST has the stacking sequence of Te-(Cr,Bi,Sb)-Te-(Cr,Bi,Sb)-Te, so-called a quintuple layer (QL), along the growth direction of [0 0 0 1] based on a hexagonal unit cell. The film thickness is basically five QL except for a small number of 1 QL high islands scattered on the surface, as demonstrated by AFM, which is not shown here. The typical growth rate is about 0.125 QL/minute. The XRD measurements were performed at the X20A beamline of the National Synchrotron Light Source at Brookhaven National Laboratory. A double Ge (1 1 1) crystal was used as a monochromator for x-rays at 8.0 keV. All measurements were carried out in air at room temperature using a standard four-circle diffractometer equipped with a Si (1 1 1) analyzer in front of a scintillation detector. The in-plane lattice constants were obtained with only one reflection so we could not specify the uncertainty. The lattice constants or thickness along the direction normal to the surface were obtained by averaging over several peaks and the error bar is the maximum deviation from the average.

## Additional Information

**How to cite this article**: Park, J. *et al*. Crystallinity of tellurium capping and epitaxy of ferromagnetic topological insulator films on SrTiO_3_. *Sci. Rep*. **5**, 11595; doi: 10.1038/srep11595 (2015).
