# Corrigendum: Crystallinity of tellurium capping and epitaxy of ferromagnetic topological insulator films on SrTiO_3_

**DOI:** 10.1038/srep14147

**Published:** 2015-09-28

**Authors:** Jihwey Park, Yeong-Ah Soh, Gabriel Aeppli, Xiao Feng, Yunbo Ou, Ke He, Qi-Kun Xue

Scientific Reports 5: Article number: 1159510.1038/srep11595; published online: 06302015; updated: 09282015

This Article contains typographical errors.

In the caption of Figure 4,

“k|| refers to the Κ-Γ-Κ direction in the reciprocal space of (Bi,Sb)2Te3 film.”

should read:

“k_//_ refers to the Κ-Γ-Κ direction in the reciprocal space of (Bi,Sb)_2_Te_3_ film.”

In the Discussion section,

“As the Te layer thickness increases, we do not detect obvious changes in the band dispersion or doping level nor do we detect a Te-derived metallic band, but the Dirac surface states of the TI gradually fade as the interface between the TI and capping layer move beyond the probe depth of our VUV ARPES experiments, which is ~1 nm below the surface.”

should read:

“As the Te layer thickness increases, we do not detect obvious changes in the band dispersion or doping level nor do we detect a Te-derived metallic band, but the Dirac surface states of the TI gradually fade as the interface between the TI and capping layer move beyond the probe depth of our VUV ARPES experiments (hv = 21.21 eV), which is ~1 nm below the surface.”

